# Taurocholic Acid Is Associated With Disturbed Functional Connectivity in the Hippocampus of Patients With Depression

**DOI:** 10.1002/advs.202508693

**Published:** 2025-12-23

**Authors:** Xiaoying Cai, Taipeng Sun, Mengzhen Feng, Gang Chen, Junchi Zhou, Hong Zhuang, Dan Wang, Ying Chen, Zhen Cheng, Zhi Xu, Xiao Zheng, Xueli Zhang, Yonggui Yuan

**Affiliations:** ^1^ State Key Laboratory of Natural Medicines China Pharmaceutical University Nanjing China; ^2^ Department of Psychiatry and Psychosomatics Zhongda Hospital School of Medicine Jiangsu Provincial Key Laboratory of Brain Science and Medicine Southeast University Nanjing China; ^3^ Department of Medical Psychology Huai'an Third People's Hospital Huaian China; ^4^ Department of Pharmacy Zhongda Hospital School of Medicine Southeast University Nanjing China

**Keywords:** bile acids, biomarker, functional connectivity, major depressive disorder, microbiome‐gut‐brain axis, taurocholic acid

## Abstract

Major Depressive Disorder (MDD) is characterized by abnormal metabolic profiles along the microbiome‐gut‐brain axis. Bile acids (BAs), a class of steroid compounds regulated by the host and microbes, are increasingly shown to become dysregulated in models of depression. However, the identity of key regulatory BA metabolite in patients with MDD and associated mechanism remain to be clarified. Here, a prospective observational study in patients with depression (*n* = 235) and control subjects (*n* = 232) for identifying functional BA metabolites regulating depressive behavior and brain functional connectivity is performed. Using comparative metabolomics assay, an increased level of taurocholic acid (TCA) in the serum of patients with MDD is observed, which is reversed by anti‐depressant treatments. Transferring fecal microbiome from patients with MDD induced TCA accumulation to the hippocampus of recipient mice exhibiting depression‐like behavior. TCA supplementation suppressed hippocampal neurogenesis, triggered microglial activation, and elicited depression‐like behavior in mice, which are alleviated by a sphingosine‐1‐phosphate receptor 2 (S1PR2) antagonist. In patients with MDD, functional neuroimaging and spearman correlation analysis revealed that circulating TCA is strongly correlated with functional connectivity in the subregions of hippocampus. The results highlight the potential of harnessing TCA as a prognostic marker and therapeutic target for depression.

## Graphical abstract

This study identifies elevated taurocholic acid (TCA) in Major Depressive Disorder patients. Gut microbiome‐associated TCA impairs hippocampal neurogenesis, triggers microglia activation, and elicits depression‐like behavior in mice via the S1PR2. In patients, functional neuroimaging reveals that serum TCA levels correlate with altered functional connectivity within hippocampal subregions. These findings position TCA as a potential prognostic biomarker and therapeutic target for depression.

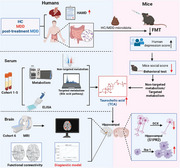

## 1 Introduction

Major depressive disorder (MDD) is a highly prevalent neuropsychiatric disease and a leading cause of disability worldwide [1]. The diagnosis of MDD largely relies on self‐rating depression scale and neuroimaging analysis, and specific biomarkers based on key pathologic events are urgently needed [2, 3]. This presents hurdles to early diagnosis and treatment of this heterogeneous disease, with more than 50% patients poorly responding to current anti‐depressants [4, 5]. Dissecting the neurophysiological basis of MDD pathogenesis and prognosis is therefore critical to solve these unmet clinical needs.

Bile acids (BAs) are a class of bioactive metabolites which are traditionally known for their regulatory roles in fat digestion and feedback signaling in the gastrointestinal tract [6]. In recent years, accumulating data from preclinical studies show that BAs and associated signaling play a critical role in brain homeostasis and behavioral control [7, 8, 9]. For example, chenodeoxycholic acid (CDCA) was shown to produce anti‐depressant effects in chronic social defeat stress (CSDS) mice via increasing the levels of phosphorylation and expression of glutamate receptors (GluA1) in the prefrontal cortex [10]. Our previous study also showed that chronic stress induced gut microbiome disturbance and brain accumulation of ursodeoxycholic acid (UDCA), which triggered depression‐like behavior in mice [8]. Of clinical relevance, abnormal BAs profile and gut microbiome disturbance have been implicated in patients with MDD [11, 12]. Also, the anti‐depressant effect of some clinical therapies has been linked to the modulation of BAs homeostasis such as DCA [13], hyocholic acid (HCA) and 7‐ketodeoxycholic acid (7‐ketoDCA) [14]. These findings suggest that deciphering the role and mechanism of BAs in MDD may provide useful biomarkers and targets for MDD.

Despite these advances and insights, several questions remain. First, whether these alterations play a causative role in patients with depression are still not fully understood. In particular, the extent to which the BA profile is linked to the anti‐depressant efficacy is not addressed by previous studies. Second, the identity of causal BA molecules in patients with MDD remains largely unknown, with previous studies from mice reporting differential impacts of BAs on depression‐like behavior [15]. Third, the neurobiological mechanisms underlying BA regulation of depression are poorly understood. For example, human neuroimaging studies have reported structural and functional network abnormalities in key brain regions under depressive states [16, 17]. However, whether BAs are correlated with some of these neuroimage changes are not understood.

In this study, we aimed to address these questions by performing a multi‐center clinical study to find potential causal BA metabolite in patients with MDD, followed by functional validation in mice. In particular, by integrating metabolomics, neuroimaging and behavioral data, we sought to identify a functional BA metabolite that could regulate brain functional connectivity and may serve as a reliable biomarker for MDD diagnosis.

## 2 Results

### 2.1 Circulating TCA is Increased in Patients with Depression and Reduced by Anti‐Depressants

**FIGURE 1:**
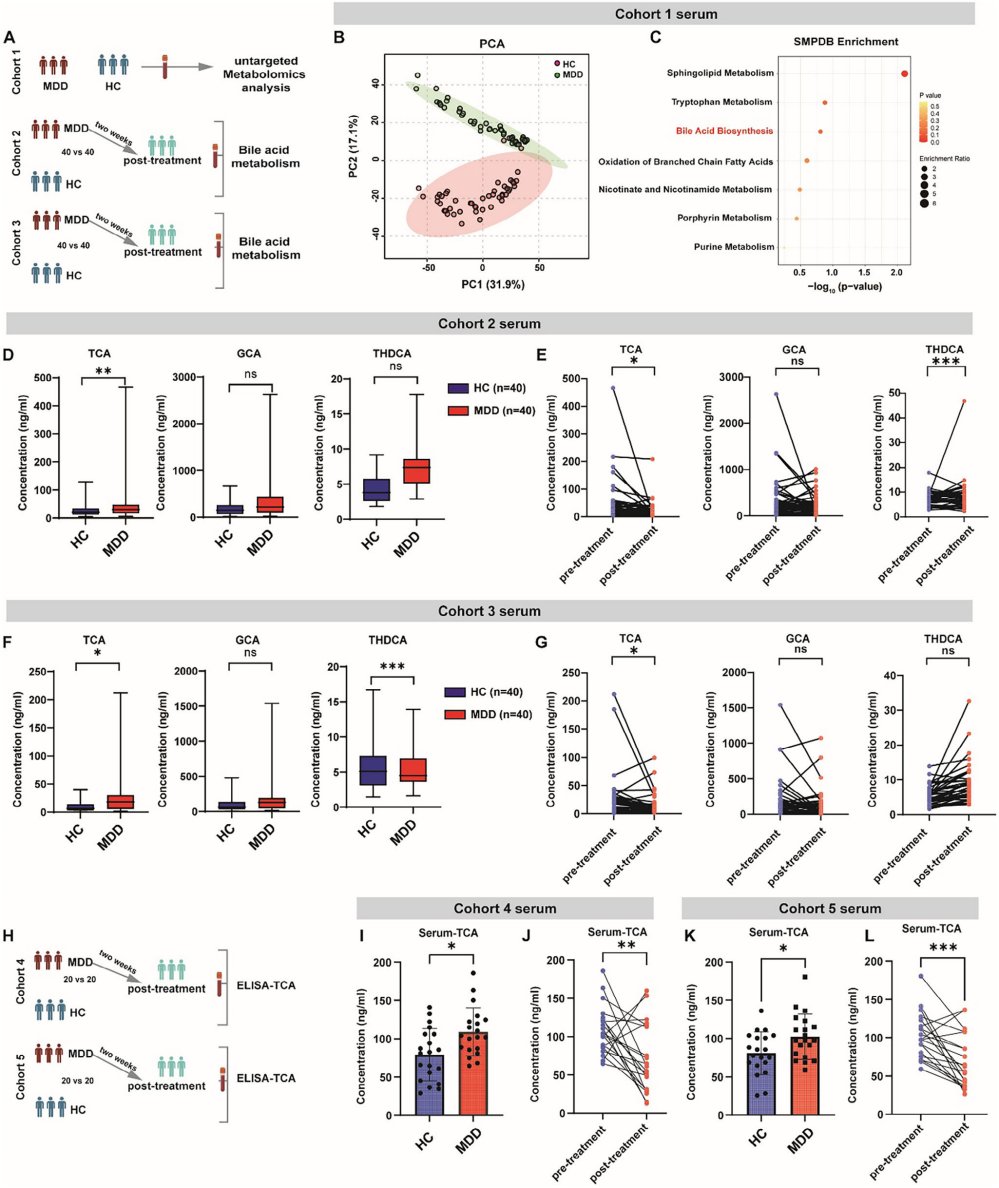
Taurocholic acid level is increased in the serum of patients with depression. (A) Schematic showing the clinical study design of cohort 1‐3 (a total of five cohorts were enrolled to collect serum samples). Serum from cohort 1 was determined by untargeted metabolomic analysis, while serum from cohort 2 and cohort 3 were subjected to bile acid‐targeted metabolomic analysis. (B) Principal components analysis (PCA) of serum metabolites from major depressive disorder (MDD) patients and healthy control (HC) in cohort 1. *p* = 0.001, R = 0.113. (*n* = 48 for HC, *n* = 46 for MDD). (B) Scatter plot of Small Molecule Pathway Database (SMPDB) metabolic enrichment analysis on the serum of patients with MDD and HC in cohort 1. (C) Box‐plot showing bile acid levels in the serum of cohort 2. TCA, taurocholic acid; GCA, glycine cholic acid; THDCA, taurohyodeoxycholic acid. *n* = 40 per group. Box‐and‐whisker plots show median and interquartile range (IQR); The whiskers above and below the box show the maximum and minimum. (D) Paired data plot showing bile acid levels by targeted metabolomic analysis of patients with MDD in cohort 2 before and after treatment. *n* = 40 per group. (E) Box‐plot showing bile acid levels by targeted metabolomic analysis between MDD and HC in cohort 3. Box‐and‐whisker plots show median and interquartile range (IQR); The whiskers above and below the box show the maximum and minimum. *n* = 40 per group. (F) Paired data plot showing bile acid levels by targeted metabolomic analysis between pre‐treatment and post‐treatment in patients with MDD in cohort 3 *n* = 40 per group. (G) Schematic showing the clinical study design of cohort 4 and cohort 5. Serum TCA levels were quantified by ELISA. (I,J) ELISA determination of serum TCA level (I), and its concentration change before and after receiving anti‐depressant treatment in patients with MDD (J) of cohort 5. *n* = 20 per group. (K, L) ELISA determination of serum TCA level (K), and its concentration change before and after receiving anti‐depressant treatment in patients with MDD (L) of cohort 6. *n* = 20 per group. *p<0.05,**p<0.01,***p<0.001; ns, no significance; Data are presented as means ± SEM (I,K) and compared by rank ANCOVA (D, F, adjusted with age, gender, and BMI) or the paired Wilcoxon rank‐sum test (E, G, J, L).

To characterize the metabolite profile alterations in MDD, we collected serum samples from 46 patients and 48 healthy individuals at Southeast University ZhongDa Hospital (Figure 1A, Cohort1). The mean age of the participants was 42.81±12.80 years, with 77.08% of them being women. Detailed demographic information is provided in Table S1. A total of 265 metabolites were structurally determined by an established untargeted metabolomics method (Figure S1A and Data S1). As expected, PCA plotting of the metabolite profiles showed a clear separation of the HC and MDD metabolite profile (Figure 1B). SMPDB analysis showed that primary bile acid biosynthesis was among the top differential pathways enriched in patients with MDD (Figure 1C; Figure S1B). To consolidate this finding, we then performed targeted analysis for on BA pathway metabolites to gain further information on BAs alterations in another cohort of patients with depression (Cohort 2, *n* = 40, Detailed demographic information provided in Table S2). Consistently, we observed an increase in the serum level of several conjugated primary bile acids (taurocholic acid, TCA), while secondary bile acids such as ursodeoxycholic acid (UDCA) remained comparable between the two groups (Figure 1D; Figure S1C).

The existence of multiple co‐varying metabolites presents a hurdle for the identification of causal player, especially in the clinical setting. We reasoned that the identification of BA metabolites that were closely affected by anti‐depressant treatment could provide more clues for functional metabolite(s) in depressive behavior. To this end, we further determined how serum BA level was affected by anti‐depressant treatment. We performed a targeted BA analysis in patients with depression from cohort 2 who received anti‐depressant treatment for two weeks, which effectively reduced the HAMD score from 27.35 ± 5.04 to 7.48 ± 4.34. We found that alleviation of depressive symptoms was specifically accompanied by decreased serum concentration of TCA but no other metabolites such as GCA or THDCA (Figure 1E; Figure S1D), suggesting the involvement of TCA in anti‐depressant action.

To explore whether this finding was robust in another center, we further recruited another cohort of patients with MDD and HCs from Huai'an Third People's Hospital (Cohort 3, *n* = 40 and 40; detailed demographic information provided in Table S3). Again, we found that serum TCA was significantly increased in patients with MDD (Figure 1F; Figure S1E). Notably, similar to the findings from the Cohort 2, TCA levels were notably reduced following anti‐depressant treatment (Figure 1G; Figure S1F). To corroborate these findings, we further recruited two cohorts of HC and patients with MDD from these two centers (Cohort 4 and 5; detailed demographic information provided in Tables S4 and S5), and determined the serum concentration of TCA by ELISA as an alternative to the LC‐MS based method. Again, we confirmed that serum TCA was significantly increased in patients with MDD but decreased after anti‐depressant treatment (Figure 1H–L). Taken together, these clinical data suggest that circulating TCA maybe functionally implicated in depressive behavior changes in humans.

### 2.2 Fecal Microbiome from Patients with MDD Induces TCA Accumulation in Mice

**FIGURE 2:**
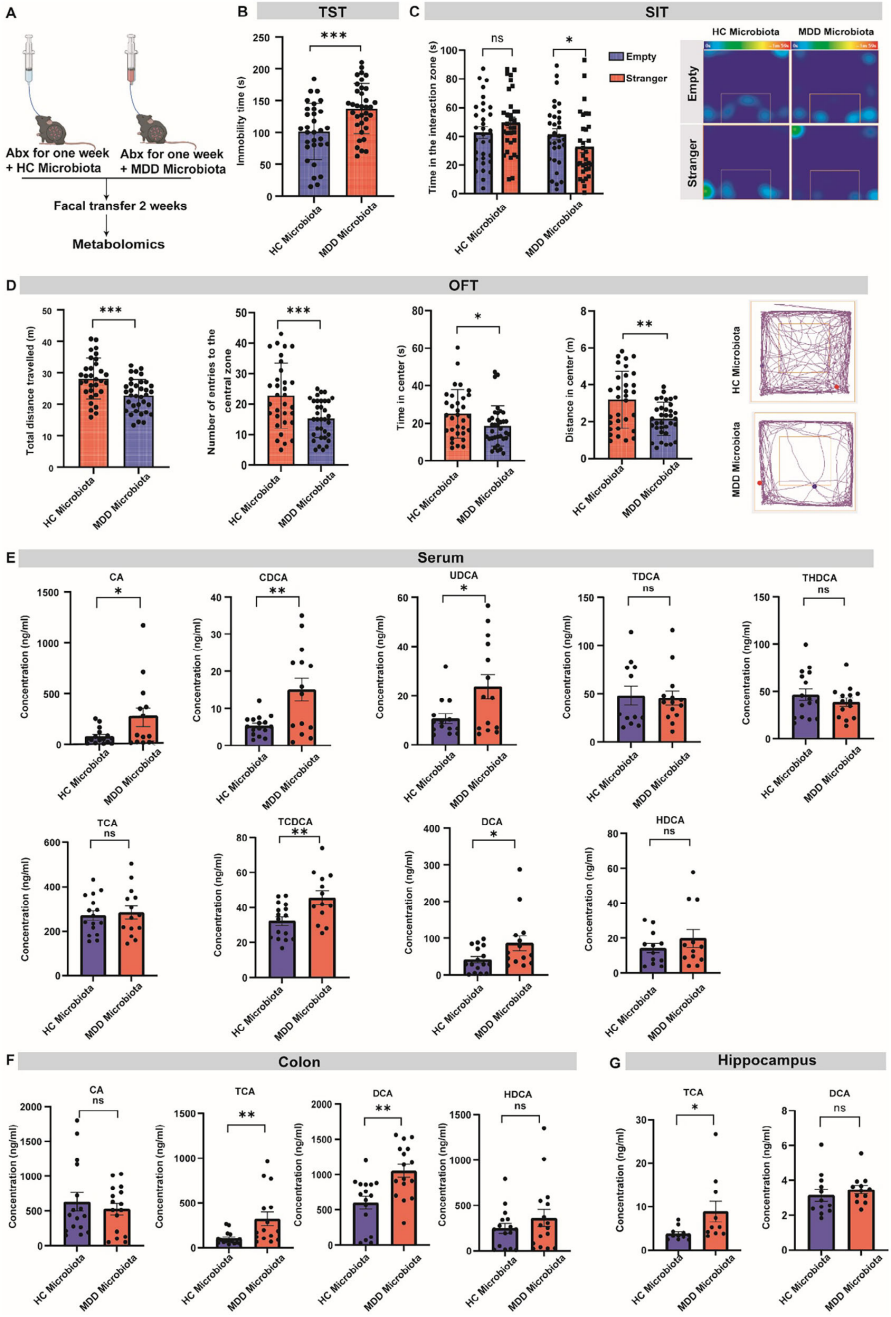
Fecal microbiome transplantation from patients with MDD induces TCA accumulation in recipient mice. (A) Schematic showing the experiment of fecal microbiome transplantation (FMT). Abx, antibiotics cocktail. (B‐D) Behavioral assessment of the recipient mice in tail suspension test (TST, B), social interaction test (SIT, C), and the open field test (OFT, D). Right: Representative SIT/OFT trajectory graph. n = 31‐35. (E) Bile acid levels in the serum of recipient mice. *n* = 12‐16. CA, Cholic acid; CDCA, chenodeoxycholic acid; UDCA, ursodeoxycholic acid; TDCA, taurodeoxycholic acid; THDCA; TCA, taurocholic acid; TCDCA, taurochenodeoxycholic acid; DCA, deoxycholic acid; HDCA, hyodeoxycholic acid. (F) Bile acid levels in the colon of recipient mice. *n* = 12‐16. (G) Bile acid levels in the hippocampus of recipient mice. *n* = 9‐12. *p<0.05, **p<0.01,***p<0.001; ns, no significance. Data are represented as mean ± SEM and compared by two‐tailed unpaired Student's *t*‐test.

BA metabolism is shaped by the gut microbiome, and gut microbial dysbiosis has been previously shown to transmit the depressive symptoms to antibiotics‐treated recipient mice [18]. We therefore further performed fecal microbiome transplant (FMT) from patients with MDD to mice and explored the changes of BA metabolites in these recipient mice (Figure 2A). We confirmed that colonization with MDD microbiota induced depressive‐like behavior in the recipient mice, as evidenced by an increased duration of immobility in the tail‐suspension test (TST, Figure 2B), a significant decrease in the time of social interaction with an unfamiliar mice in the social interaction test (SIT, Figure 2C), as well as significantly fewer entries, duration, and distance in the center zone in the open‐field test (OFT, Figure 2D).

We observed a clear separation of the serum metabolite profiles in recipient mice for the HC and MDD microbiota (Figure S2A; detailed differential metabolites information provided in Data S2). SMPDB analysis also showed a major difference on the primary bile acid biosynthesis pathway (Figure S2B). We further performed targeted analysis of BAs in the serum, colon, and hippocampus of recipient mice. Although serum TCA was not significantly changed (Figure 2E), it was consistently increased in the colon and hippocampus of MDD‐microbiota recipient mice (Figure 2F,G). Bacterial *16S* rRNA sequencing of cecal samples confirmed that FMT induced a distinct microbial configuration in the recipient mice (Figure S2C,D). PICRUSt (Phylogenetic Investigation of Communities by Reconstruction of Unobserved States) prediction based on the sequencing data indicated a significant decrease in bile acid biosynthesis pathway in MDD‐microbiota recipient mice (Figure S2E). Together, the increase of TCA in the gut and brain of recipient mice after FMT further supports a close association of this metabolite with depressive behavior.

### 2.3 TCA Triggers Depression‐Like Behavior in Mice

**FIGURE 3:**
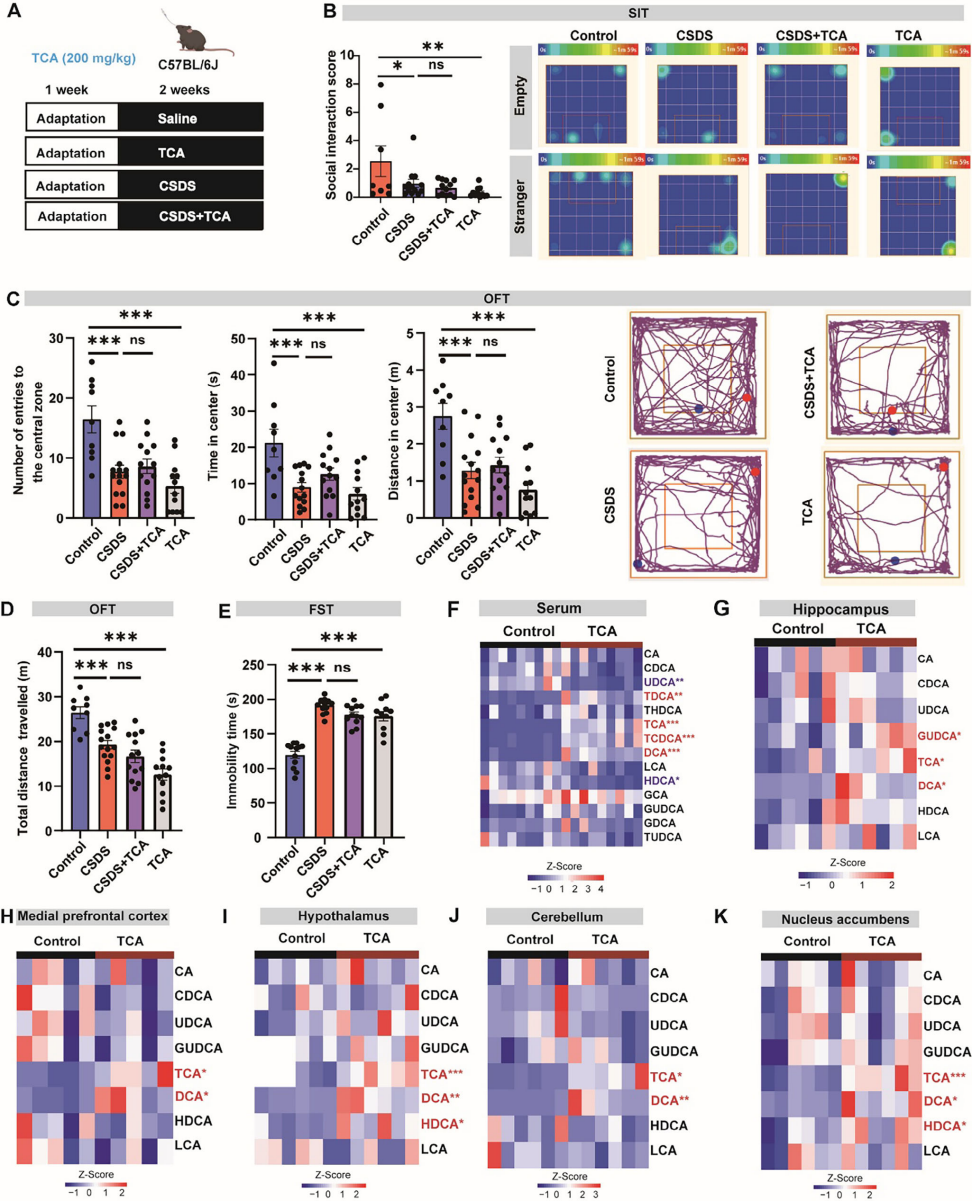
TCA triggers depression‐like behavior in mice. (A) An overview of the study of TCA treatment to CSDS mice models. TCA was administered by oral gavage at 200 mg kg^−1^ for two weeks. (B–E) Behavioral assessment of mice in social interaction test (SIT, B), the open field test (OFT,C,D), and forced swimming test (FST, E). *n* = 9‐15. (F–K) Heatmap showing the changes in bile acid levels in the serum (F), hippocampus (G), medial prefrontal cortex (H), hypothalamus (I), cerebellum(J), and nucleus accumbens (K) between control group and TCA supplemented group. *n* = 5–9 per group. Bile acids showing significant changes were highlighted in red. *p<0.05,**p<0.01,***p<0.001; ns, no significance. Data are represented by mean ± SEM, and compared by One‐way ANOVA (B‐E) or two‐tailed unpaired Student's *t*‐test (F–K).

To directly determine the impact of TCA on depressive‐like behavior, based on previous report and our preliminary study [19], we tested the behavioral effects of TCA supplementation (200 mg kg^−1^) to control and chronic social defeat stress (CSDS)‐induced depressive mice (Figure 3A). Notably, we observed a clear appearance of depression‐like behavior in control mice supplemented with TCA, as evidenced by decreased social interaction score in the SIT (Figure 3B), decreased exploration of the central area during OFT (Figure 3C,D), increased immobility in the FST (Figure 3E). Nevertheless, we found that TCA supplementation to CSDS mice did not exacerbate the core symptoms of depression in a series of behavioral tests (Figure 3B–E). Previous studies indicated that TCA could cross the blood‐brain barrier largely via active transport [20, 21]. In consistence, we confirmed the expression of related influx and efflux transporters in the hippocampus of mice (Figure S3A). We verified that oral administration of TCA significantly increased its concentration in the serum and brain regions such as the hippocampus, hypothalamus and prefrontal cortex of mice, suggesting a direct action of TCA in the brain (Figure 3F–K). These findings collectively indicate that TCA could enter the brain to trigger depressive‐like behavior in mice.

### 2.4 S1PR2 Antagonist Blocks TCA‐Induced Depressive‐Like Behavior

**FIGURE 4:**
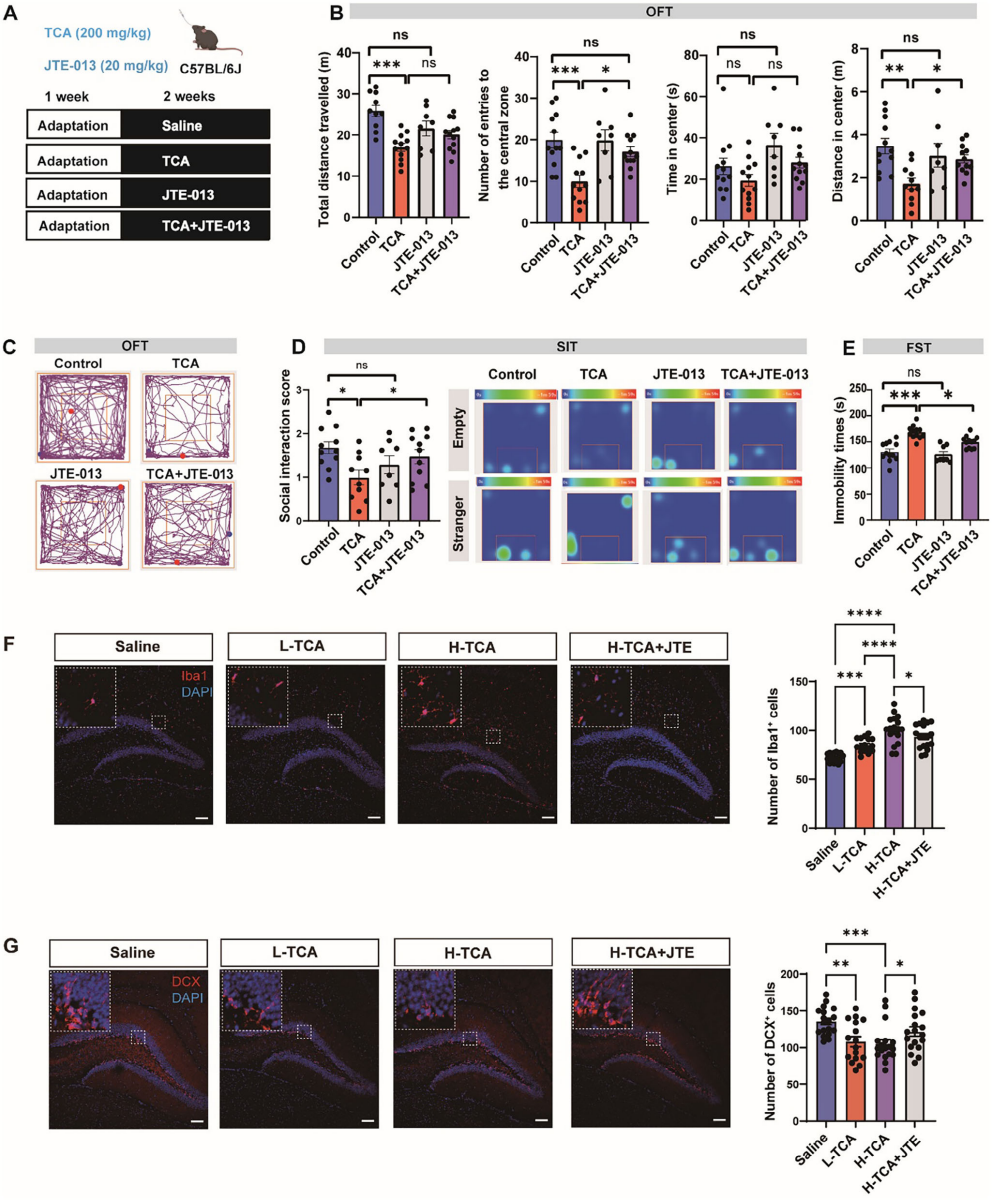
S1PR2 antagonist JTE‐013 blunts the behavioral effects of TCA in mice. (A) Schematic showing of the experiments with S1PR2 antagonist JTE‐013 in mice. (B–E) Behavioral assessment of mice in the open field test (OFT, B,C), social interaction test (SIT, D), and forced swimming test (FST, E). *n* = 8–12. (F) Representative immunofluorescence images of Iba‐1 staining in the hippocampus, and the comparison of Iba‐1^+^ cells in each slices derived from the brain of 6 mice. Typical morphology of microglia are zoomed in. Scale bars:100 µm. Mice were orally treated with L‐TCA (100 mg kg^−1^) or H‐TCA (200 mg kg^−1^). (G) Representative immunofluorescence images of DCX staining in the hippocampus, and the comparison of DCX^+^ cells in each slices derived from the brain of 6 mice. Typical images of neurons are shown in the inserts. Scale bars:100 µm. Mice were orally treated with L‐TCA (100 mg kg^−1^) or H‐TCA (200 mg kg^−1^). *p<0.05,**p<0.01,***p<0.001; ns, no significance. Data are represented as mean ± SEM, and compared by One‐way ANOVA.

It was previously reported that TCA can interact with the sphingosine 1‐phosphate receptor 2 (S1PR2) to promote neuroinflammation during hepatic encephalopathy in mice [22]. By PCR analysis, we confirmed an extensive expression of *S1pr2* in major brain regions including the cerebellum, hypothalamus, prefrontal cortex, hippocampus, and nucleus accumbens (Figure S3B). TCA supplementation did not affect the mRNA expression of *S1pr2* in these brain regions (Figure S3C). To explore whether S1PR2 was functionally implicated in the pro‐depressive effects of TCA, we treated mice with a brain‐permeable S1PR2 antagonist JTE‐013 [23](Figure 4A). We found that JTE‐013 alone did not affect the performance of control mice in the OFT, FST, or SIT (Figure 4B–E). Nevertheless, S1PR2 blockade by JTE‐013 effectively negated the behavioral impacts of TCA, as indicated by increased central entries in the OFT and social interaction score of TCA‐treated mice (Figure 4B–E), suggesting the implication of S1PR2 signaling to the pro‐depressive effects of TCA in mice.

S1PR2 was previously implicated in neuroinflammation [24, 25]. To understand how TCA/S1PR2 axis triggered depressive‐like behavior in mice, we further examined the status of microglia activation using ionized calcium binding adapter molecule 1 (Iba‐1) as a canonical markers. As expected, we observed that there was a significantly increased density of Iba‐1^+^ microglia in the hippocampus of TCA treated mice, accompanied by a prototypical morphology of activated microglia (Figure 4F). In contrast, JTE‐013 treatment significantly reduced the number of Iba‐1^+^ cells, which also displayed a less activated status (Figure 4F). Since neuroimmune activation is known to impair neurogenesis, next we explored the changes in neurogenesis, the impairment of which is well implicated in depressive‐like behavior [3]. In consistence, by doublecortin (DCX) staining, we found that TCA induced a clear impairment of hippocampal neurogenesis (Figure 4G). Notably, we observed a significant alleviation of neurogenesis impairment in JTE‐013 treated mice, indicating that TCA activated S1PR2 to trigger neuroinflammation and neurogenesis impairment in mice.

### 2.5 TCA is Associated with Functional Connection Abnormality in the Hippocampal Subfields of Patients with MDD

**FIGURE 5:**
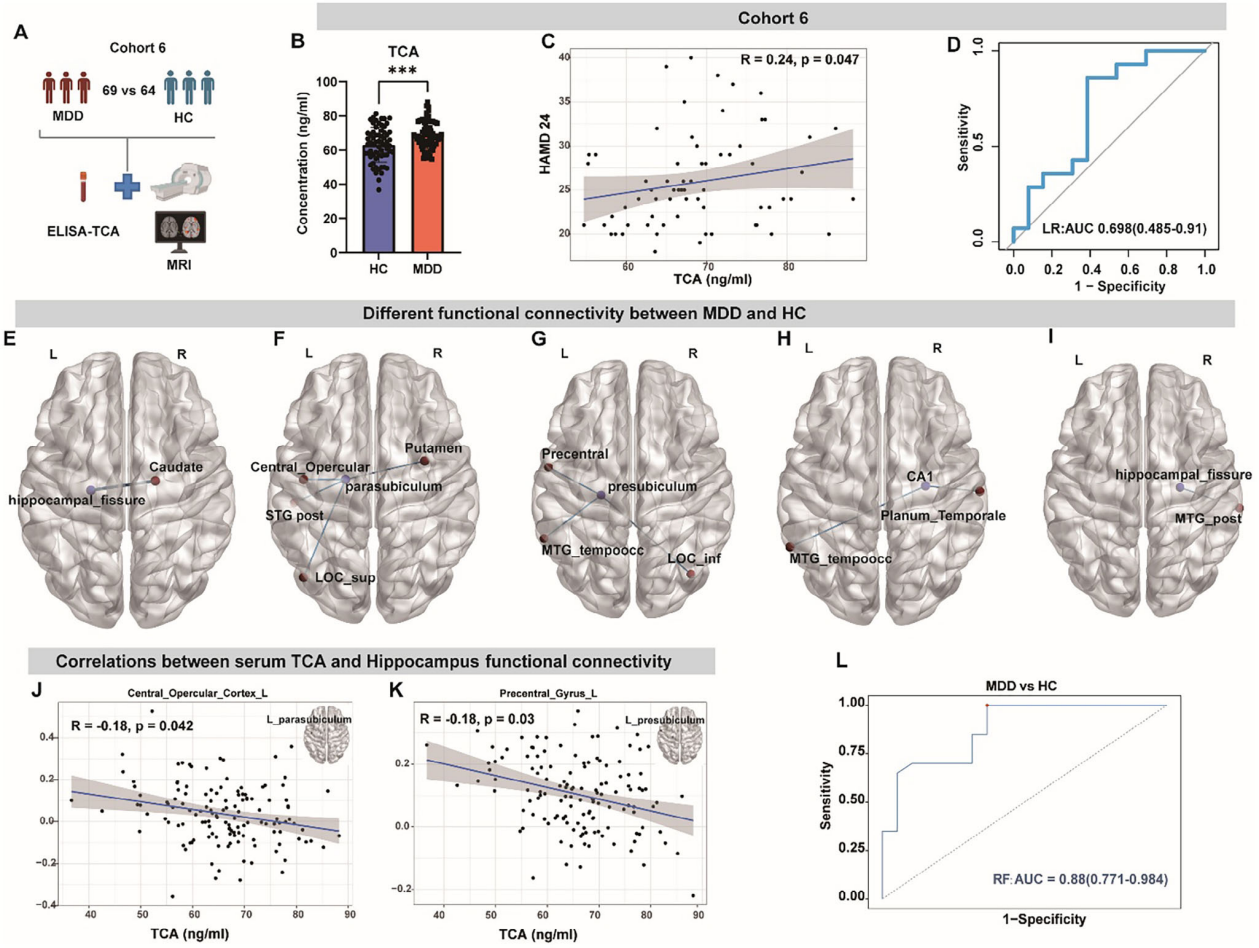
Serum TCA level is associated with functional connectivity of the hippocampal subfields to whole brain. (A) An overview of clinical validation study design. (B) Serum level of TCA between MDD (*n* = 69) and HC (*n* = 64) quantified by ELISA in cohort 6. (C) Spearmen's correlation between serum TCA level and HAMD‐24 score in patients with MDD. (D) Receiver Operating Characteristics (ROC) generated between MDD and HC by TCA based on logistic regression model. (E–I) Different functional connectivity between MDD and HC based on seed‐whole‐brain functional connectivity analyses. (E, left hippocampal_fissure; F, left parasubiculum; G, left presubiculum; H, right CA1; I, right hippocampal_fissure), with blue indicating lower in patients with MDD (adjusted for age and gender). (J, K) Significant association between serum level of TCA and functional connectivity in two hippocampal subfileds. (L) ROC generated between patients with MDD and HC by TCA and 5 functional connectivity selected by the lasso regression based on random forest classification model. ***p<0.001; ns, no significance; Data are compared by two‐tailed ANCOVA adjusted with age, gender, and BMI.

The findings above from mice prompted us to explore the potential prognostic value of TCA for MDD in the clinic. To this end, we recruited another independent cohort of participants (Cohort 6, *n* = 69 and 64, Figure 5A). There were no significant differences between the two groups in terms of age, gender, and BMI (Table S6). After controlling for the age, gender, and BMI, we observed that the level of TCA in serum was consistently higher in the MDD group (Figure 5B). We also observed a positive correlation between TCA and HAMD‐24 scores (Figure 5C). In contrast, no significant correlation between TCA and HAMA was found (r = 0.12, *P* = 0.31; Figure S4A). ROC analysis of TCA showed promising discrimination power between MDD and HC cohorts (Figure 5D).

Given that TCA could disturb hippocampal neurogenesis in mice, which was closely associated with synaptic function, we further examined the functional connection (FC) in the hippocampal subfields to investigate the correlation between serum TCA levels and hippocampal function. To this end, we performed MRI imaging in a cohort of the patients with MDD and healthy subjects, and compared FC by controlling for the age, gender, and BMI. Compared to HC, patients with MDD showed decreased FC with the left hippocampal_fissure and right caudate (Figure 5E). In addition, patients with MDD showed significantly decreased FC between left parasubiculum and left Central Opercular Cortex, left Lateral Occipital Cortex (superior division, LOC_sup), left Superior Temporal Gyrus (posterior division, STG_post), right Putamen (Figure 5F). Patients with MDD also had significantly decreased connectivity of left presubiculum with left Middle Temporal Gyrus (temporooccipital part, MTG_temporoocc), right Lateral Occipital Cortex (inferior division, LOC_inf), left Precentral Gyrus (Figure 5G), and significantly decreased connectivity between right CA1 and left MTG_temporoocc, right Planum Temporale (Figure 5H). Patients with MDD also had significantly decreased FC between right hippocampal_fissure and right Middle Temporal Gyrus (posterior division, MTG_post; Figure 5I). These data indicate that the FC of five hippocampal subregions with whole brain were different between MDD and HC subjects. Details on these regions with between‐group FC differences are listed in Table S7.

We further determined the correlation of serum TCA level with FC changes in major brain regions. Spearman's correlation analysis revealed negative correlations between TCA and FC in two brain regions: specifically, between the left parasubiculum and the left Central Opercular Cortex (Figure 5J), and between the left presubiculum with the left Precentral Gyrus (Figure 5K). To further explore the predictive values, feature down‐selection was performed using the LASSO method, and then five brain functional characteristics were selected to construct a random forest model (Figure S4C; Figure S4B). The random forest classification model based on TCA and five brain functional characteristics achieved a higher AUC of 0.88 (Figure 5L). In addition, feature importance descriptors quantified based on random forest model indicated that FC between the left parasubiculum and the right Putamen was the most important (Figure S4D). Together, these data indicate that increased TCA in patients with MDD may affect FC abnormality in the hippocampal subregions to modulate depressive behavior.

## 3 Discussion

MDD is a psychosomatic disease with heterogeneous pathophysiological mechanisms. Identifying causal metabolite markers and associated mechanisms are promising to guide precision diagnosis and treatment of this detrimental mental disease [26]. In this study, we report that increased level of circulating TCA could serve as a reliable biomarker for depressive behavior. Notably, TCA level was decreased by anti‐depressant treatment, and its supplementation triggered depressive‐like behavior in mice, which was partially attributed to microglia activation and neurogenesis impairment. In patients with MDD, circulating TCA showed a strong correlation with FC in hippocampal subregions. These findings highlight the potential of harnessing circulating TCA as a prognostic marker and therapeutic target for depressive disorder.

Disturbance of BA metabolites is commonly observed in the serum of patients with depression and animal models [11, 27, 28], yet the causative role of key metabolites is largely elusive. In order to find the functional metabolite more effectively, here we profiled BA changes in two cohorts of patients with MDD both at baseline and after anti‐depressant treatment. The significant decrease in serum TCA accompanying the decreased HAMD score suggests a close association with the depressive symptoms. However, for some other BA metabolites such as GCA and THDCA, we did not observe such a consistent change in the different cohorts, suggesting that their relationship to depressive behavior is not so robust as TCA. It is interesting to note that only TCA exhibited a significant reduction following anti‐depressant treatment. The potential mechanisms for the selective downregulation of TCA are not addressed by current study, but most likely involve the modulation of gut microbes that affect the conjugation/deconjugation metabolism of TCA. The serum concentration of TCA is determined by several factors, mainly including hepatic synthesis and microbial hydrolysis to primary bile acids [8, 29, 30]. In our study, we observed that FMT from patients with MDD induced TCA accumulation to the colon and brain of recipient mice, which indicated that gut dysbiosis directly contributed to disturbed TCA metabolism. Since the *16S* rRNA sequencing data revealed the alteration of multiple bacterial genera, future studies are needed to understand the key bacterial species that contribute to TCA accumulation and whether promoting its hydrolysis would help improve the behavioral abnormalities. Although we show that supplementation of TCA could disturb hippocampal neurogenesis and induce depression‐like behavior in mice, it could also reflect a downstream consequence of depression. Indeed, chronic psychological stress and depression‐like behavior are known to disturb bile acid metabolism and the gut microbiome, which could in turn affect TCA level.

BAs are known to exert neuroimmune regulation via interaction with dedicated receptors such as farnesoid X receptor (FXR) and Takeda G‐protein‐coupled receptor 5 (TGR5) [31]. In our study, we focused on the role of S1PR2 for previous knowledge on its affinity for TCA and role in mediating the inflammatory effect of TCA in the liver and brain [22, 32, 33]. We confirmed the role of S1PR2 by showing that the depressive‐like behavior of TCA treated mice was effectively improved by a brain‐permeable antagonist of this receptor. As for the regulation of S1PR2 by stress, a previous study showed that S1PR2 protein expression in hippocampus was reduced in chronic unpredictable stress exposed rats [34]. This might partially explain why no additive effect of TCA on the CSDS model was observed in our study, and future studies are warranted to explore the exact reason. Moreover, our data indicated that the behavioral change may be attributed to improvement of hippocampal neurogenesis and microglial activation, which are key mediators of behavioral change in depression [35, 36]. Of interest, supporting our finding, a recent study also reported that TCA induced synaptic loss in vitro, which may contribute to cognitive impairment in elderly individuals [37]. However, our data cannot exclude the contribution of other BA receptors such as TGR5 and other brain regions with S1PR2 expression. Indeed, TGR5 in the lateral hypothalamus (LHA) influences neuronal dynamics underlying stress‐induced depression‐like behaviors [9]. LHA GABAergic TGR5 exerted antidepressant‐like effects by disinhibiting dCA3 CaMKIIα neurons projecting to the dorsolateral septum (DLS) [9]. Therefore, given the exposure to multiple BAs in different neural circuits and brain regions, it would be interesting to propose that TCA may simultaneously activate mutually antagonistic TGR5/S1PR2 pathways to induce depressive behavior. Future studies are warranted to find the target cell and receptors that may contribute global insights into the action of TCA in the brain.

In our previous studies and others, it is observed that BA metabolism is disturbed in mice models of depression and patients with depression [8, 38]. Nevertheless, there are still limited insights into a reliable biomarker that enable sensitive and reliable diagnosis of depression for clinical translation. We therefore further explored the potential relationship between serum TCA and functional MRI data in the brain, which proved useful to aid clinical diagnosis of depression [39, 40]. We focused on the hippocampal subregions based on the TCA distribution and neurogenesis data from animal study, although the contribution of many other brain regions cannot be ignored. Importantly, TCA showed negative correlations with the FC between the left parasubiculum and the right Putamen, which showed the most notable contribution to the differentiation between patients with MDD and HC individuals. This finding indicated that TCA may play a role in shaping the crosstalk between these two regions with well confirmed role in affective behavior, and the specific mechanism especially from the involvement of S1PR2 remains to be determined in future studies.

## 4 Conclusions

In conclusion, by combining metabolomics, brain imaging and behavioral data from clinical patients and mouse model, here we report that serum TCA may serve as a novel biomarker for depressive disorder partially attributed to its role in shaping functional connectivity of hippocampal subregions. Mechanistically, TCA accumulation could be partially attributed to gut dysbiosis and its pro‐depressive effect depends on S1PR2. Our findings in mouse model and human suggest that reducing TCA levels in the serum by promoting its hydrolysis or inhibiting its receptor S1PR2 may offer new possibilities in designing therapeutic strategies for depressive disorder.

## 5 Experimental Section

### 5.1 Human Participants

The authors assert that all procedures contributing to this work comply with the ethical standards of the relevant national and institutional committees on human experimentation and with the Helsinki Declaration. All procedures involving human subjects/patients were approved by the Ethics Committee of the Affiliated ZhongDa Hospital of Southeast University (2022ZDSYLL446‐P01). In total, six cohorts were recruited from ZhongDa Hospital and Huai'an No. 3 Hospital. Diagnosis of MDD was conducted based on DSM‐IV criteria by two senior psychiatrists, and age‐ and gender‐matched healthy controls (HC) were recruited from the two centers for a prospective study. The inclusion criteria for MDD group were as follows: 1) fulfillment of the DSM‐IV criteria for MDD; 2) aging from 18 to 65 years old; 3) right‐handedness; 4) HAMD‐24 total score ≥ 20. The exclusion criteria for MDD group were as follows: 1) any other neurological or psychiatric diseases; 2) any major medical conditions; 3) metal implants (due to MRI contraindications); 4) structural or signal abnormalities of intracranial lesions in MRI scans; 5) pregnant or breastfeeding; 6) had used antibiotics in the past 3 months. The inclusion criteria for HC group were: 1) age and gender were matched with depression patients; 2) no history or family history of any mental illness. Exclusion criteria for the HC group were similar to those for the MDD group. Finally, 6 cohorts (including 237 patients with MDD and 232 control subjects) were enrolled for serum samples, and cohort 6 (including 69 patients with MDD and 64 HC participants) was additionally included for MRI scans. All participants provided written informed consent. Depression and anxiety were assessed using the Hamilton Depression Rating Scale‐24 items (HAMD‐24), and the Hamilton Anxiety Rating Scale (HAMA). Snaith‐Hamilton Pleasure Scale (SHAPS) was used to assess the anhedonia.

In order to explore the impact of anti‐depressants treatment on BAs, all patients were required to be free of any psychotropic medications for at least two weeks prior to enrollment. After enrollment, patients in cohorts 2‐5 all began to receive initial treatment with selective serotonin reuptake inhibitors (SSRIs), and the specific SSRI drugs used were shown in Table S3.The primary outcome assessment was conducted two weeks after the initiation of treatment, during which HAMD‐24 scores and BA levels were measured for all patients.

### 5.2 Mice and Treatment

Male C57BL/6J mice (6‐week, 20 g) were obtained from Vital River Experimental Animal Technology Co. Ltd (Beijing, China). All the mice were housed in a standard specific‐pathogen‐free (SPF) conditional room (12 h light:12‐h dark cycle, room temperature 22 ± 2 °C), with food and water provided ad libitum. All the mice were allowed to adapt for 1 week in the housing condition before the initiation of experiment. All the mouse studies were approved by the Animal Ethics Committee of China Pharmaceutical University (2022‐05‐037) and strictly followed the ARRIVE guidelines.

To explore the dependence of S1PR2 in the action of TCA, mice were randomly divided into four groups: 1) Control group (*n* = 11), 2) TCA group (200 mg kg^−1^, *n* = 12), 3) TCA + JTE‐013 group (20 mg kg^−1^, *n* = 12), 4) JTE‐013 group (20 mg kg^−1^, *n* = 8). The dosage of JTE‐013, a brain permeable S1PR2 antagonist, was based on previous reports [23].

### 5.3 Fecal Microbiome Transplant

Fecal microbiome transplant (FMT) was performed based on an established protocol previously described by us and others [18]. Briefly, recipient mice were given antibiotic cocktails (6.7 mg mL^−1^ of ampicillin, 6.7 mg mL^−1^ of metronidazole, 6.7 mg mL^−1^ of neomycin, 3.35 mg mL^−1^ of vancomycin) by gavage for 10 days. FMT was performed after a wash‐out period for three days. Stool samples were selected from 4 HC individuals and 4 unmedicated patients with MDD (detailed information provided in Table S8), who provided feces samples on the day of admission. The stool samples were snap frozen and stored at −80 °C within 0.5 h. Aliquots of thawed stool samples were freshly resuspended in sterile pre‐reduced PBS at the same proportions (15 mL per gram of feces), vortexed for 5 min and centrifuged at 3000 rpm for 5 min. Equal volumes of the supernatant from each group were combined, and an equal volume of sterile 40% (w/v) glycerin was added for preservation. The mixture was deposited separately and gavaged into ABX‐treated mice (*n* = 8 for each donor fecal sample) every other day (200 µL per mouse) for 2 weeks.

### 5.4 Chronic Social Defeat Stress in Mice

Before the experiment, retired CD‐1 breeder mice (Vital River Experimental Animal Technology Co. Ltd) were screened to be qualified aggressive mice and then housed singly in one side of cage equipped with perforated divider. To induce chronic social defeat stress (CSDS), the C57BL/6 mice were exposed to novel CD‐1 aggressors for 10 consecutive days. During daily social confrontation (between 16:00 pm to 18:00 pm), a C57BL/6 mouse was transferred to the homecage of an aggressive CD‐1 mouse for 10 min to receive sustained defeat. After that, the “intruder” was transferred to the other side of the divider and housed overnight. Care was taken to maximally avoid any physical lesions on the “defeated” mice. Control mice were left in their home cages undisturbed.

### 5.5 Behavioral Assessment

*Open‐Field Test (OFT)*: After acclimation to the test environment for at least 1 h, mice were gently placed in the center of a white plastic open‐field arena (40 cm × 40 cm × 40 cm) and allowed to explore freely for 8 min. A video camera positioned directly above the arena was used to track the movement of each animal, and recorded by ANY‐maze software (Stoelting) to track the total distance and the amount of time spent in the center and periphery of the chamber. The arena was cleaned with 75% ethanol after every trial session.

### 5.6 Tail Suspension Test (TST)

After acclimation to the test environment for at least 1 h, mice were suspended by their tails with tape in a position that they could not escape for 6 min. The total duration of the test can be divided into periods of agitation and immobility. Higher degrees of depression are indicated by less time spent trying to escape in the last 4 min.

### 5.7 Social Interaction Test

Each social interaction test (SIT) was composed of two 150 s phases, separated by a duration of 30 s, either with or without the target CD‐1 mouse present in the interaction zone [18]. During the first phase, when the target CD‐1 aggressor was absent, the C57BL/6J mouse was placed directly into the rear center of the open field opposite to the empty wire mesh enclosure, allowing for free exploration of the open‐field arena. Immediately after 150 s, the C57BL/6J mouse was removed from the arena and returned to its home cage until phase 2. Again, the C57BL/6J mouse was removed from its home cage and placed into the rear center of the open field opposite the wire‐mesh enclosure. A video camera positioned directly above the arena was used to track the movement of each animal, and ANY‐maze software was employed to track the amount of time spent in the interaction zone during the absence and presence of the CD‐1 mice. The arena was cleaned with 75% ethanol after every trial.

### 5.8 Immunofluorescence Analysis of Hippocampal Samples

Mice were deeply anesthetized by inhalation of isoflurane and perfused transcardially with 4% paraformaldehyde (PFA). Brains were post‐fixed in 4% PFA at 4 °C overnight, then cryoprotected in 30% sucrose overnight. Coronal slices (12 µm thick) were obtained from frozen tissue using a sliding blade microtome then transferred to ice cold PBS. Slices were blocked with 10% normal donkey serum, 0.3% Triton X‐100 0.1 M PB (PBTgs) for 1 h at room temperature and then incubated in rabbit anti‐doublecortin (DCX) primary antibodies (Proteintech, Cat #: 13925‐1‐AP, 1:200), or anti‐Iba‐1 primary antibodies (Abcam, Cat #: ab178847, 1:200) at 4 °C for 18 h. Donkey anti‐rabbit Alexa Fluor 555 (Abcam, Cat #: ab150062, 1:500) were used as secondary antibodies. Finally, the cell nuclei were stained with 4,6‐diamidino‐2‐phenylindole (DAPI). Fluorescent imaging and data acquisition were performed on an LSM700 microscope (ZEISS) using ZEISS ZEN software. Images were analyzed in Fiji/Image J and backgrounds were kept the same for different groups. A positive staining is guided by the obvious shape of an individual neuron. Image processing was applied uniformly across all images within a given dataset. In the hippocampal denticulate gyrus (DG) region of each mouse (*n* = 6), 2 to 3 sections (30 µm) were obtained and analyzed. The number of DCX/Iba‐1 positive cells in each section was analyzed based on the morphology of neurons or glial cells.

### 5.9 Metabolomics Assay in Clinical Serum Sample

The venous blood of the participants was collected on the next day after admission. Participants were well informed to refrain from eating for a minimum of 8 h prior to specimen collection in order to avoid the influence of food on metabolic status. Peripheral venous blood was drawn into heparinized tubes, and centrifuged at 1800 g for 10 min. Serum samples were frozen at −80 °C until further metabolomics analysis by an established protocol [3]. Briefly, metabolomics analysis was performed on an ultra‐high‐performance liquid chromatography coupled to mass spectrometry (UPLC‐MS). Serum samples were prepared by protein precipitation with methanol. The supernatant was dried using a vacuum concentrator, and reconstituted in 150 µL of cold methanol and vortexed for 15 min before HPLC injection.

Chromatographic separation was achieved using a Waters ACQUITY UPLC HSS T3 column (2.1 × 100 mm, 1.8 µm, Waters), maintained at a temperature of 40 °C. The mobile phase consisted of 5 mM ammonium formate with 0.5% formic acid (Phase A) and acetonitrile (Phase B), which eluted following a gradient program. The metabolites were detected in the positive ion modes using electrospray ionization (ESI). MS parameters were set as follows: TOF‐MS scan, m/z 50‐1000 Da; product ion scan, m/z 50‐900 Da; the drying gas temperature was set at 550 °C (ESI +); the ion spray voltage was + 5500 V (ESI +); the collision energy (CE) as set at +40 V (ESI +). Ion source gas 1 (gas 1), ion source gas 2 (gas 2) and curtain gas in both ionization modes, were set at 50, 30, and 30 psi, respectively. The data processing was done by Progenesis QI (version 2.0, Waters). Preliminary search was performed by MetaScope to identify possible metabolites. Subsequently, eluents with MS/MS data underwent fragment match based on a local version of METLIN Library, which was applied as a plugin for QI. Search parameters for MetaScope were set as follows: precursor tolerance (10 ppm); retention time within (0.1 min); and fragment tolerance (15 ppm). Parameters for METIN were set as: precursor tolerance (10 ppm), and fragment tolerance (15 ppm).

For the preprocessing of non‐targeted metabolomics data, features with a missing value ratio exceeding 33% are deleted. When there are zero values in the data, the default detection limit method (LoD, detection limit) will use 1/5 of the minimum positive value of each feature as an alternative value for the detection limit to fill in all the missing values. Normalization method: Normalization by sum; Data transformation method: None; Data scaling method: None. Pathway analysis was performed through metabolite set enrichment analysis by online analysis software. The specific steps are as follows: Log in to the official website https://www.metaboanalyst.ca/, click the button “click here to start”, select the “enrichment analysis” module, fill the first column of the difference metabolite table with the names of the difference metabolites in the “compound list”, select “Metabolites” for Feature Type, click “submit”, click “proceed”, select “SMPDB” for pathway based, click “submit”, and you will obtain the difference enrichment pathways.

### 5.10 Targeted Analysis of BAs by LC‐MS

The determination of BA metabolites in clinical and mice samples was performed following the procedure as described previously [8]. Briefly, targeted analysis was performed using ABSciex Q‐TRAP 5500 mass spectrometry (Foster City, CA, USA). The separation was performed on a Waters Atlantis T3 column (100 mm × 2.1 mm, 3.0 µm, Waters, USA). The mobile phase (solvent A) was 0.1% formic acid aqueous solution, and the organic phase (solvent B) was acetonitrile. The column temperature was 40 °C and the flow rate was 0.3 mL min^−1^ with gradient elution. The mass spectrometry parameters were set as the same with previous report [8].

### 5.11 Quantitative Real‐Time PCR

Total RNA was extracted from mouse brain tissue using RNAiso Plus reagent (Vazyme). RNA samples were reverse transcribed to cDNA using PrimeScript RT Reagent kit (TransGen). Real‐time PCR amplification was performed using SYBR Green Supermix (Bio‐Rad). The target gene was normalized to *Gapdh* and expressed as fold change. The primer sequences were shown in Table S9.

### 5.12 Enzyme‐Linked Immunosorbent Assay

Taurocholic acid (TCA) concentration in the serum of patients was detected by a commercially available enzyme‐linked immunosorbent assay (ELISA) Kit (Cat# CEO267Ge) with confirmed linearity, sensitivity and precision. Samples were measured in duplicate according to the manufacturer instructions. After adding 50 µL of termination solution to each well, the absorbance was immediately detected by a microplate reader (BioTek) at 405 nm.

### 5.13 MRI Acquisition

All participants underwent imaging in a 3.0 Tesla whole‐body MRI scanner (Siemens Medical Systems, Erlangen, Germany) for a high‐resolution T1 structural scan, and a resting‐state functional scan. The acquisition parameters were as follows: T1‐weighted magnetization‐prepared rapid gradient echo (MPRAGE) scans acquired to assess brain structure, repetition time (TR) = 1900 ms; echo time (TE) = 2.48 ms, flip angle (FA) = 9°, acquisition matrix = 256 × 256, field of view (FOV) = 250 × 250 mm^2^, slice thickness 1.0 mm, and volume = 176. Resting‐state fMRI scans were acquired to assess resting‐state functional connectivity (FC), TR = 2000 ms; TE = 25 ms, FA = 90°, acquisition matrix = 64 × 64, FOV = 240 × 240 mm^2^, slice thickness = 3.0 mm, axial slice = 32, volume = 240; in‐plane resolution parallel to the anterior‐posterior conjunction = 3.75 × 3.75 mm.

### 5.14 Functional Image Data Processing

The rs‐fMRI images were preprocessed by the DPABI toolbox (http://rfmri.org/dpabi). The whole process included the removal of the first 10 Echo Planar Imaging (EPI) scans, correction of slice timing, head motion (excluded subjects with maximum motion >2.5 mm), spatial normalization, nuisance signal regression, data scrubbing, spatial smoothing, and band‐pass filtering (0.01–0.1).

Based on the finding from our animal studies, we chose hippocampal subfields as the primary ROIs to investigate the correlation between serum TCA levels and hippocampal function using MRI data. Segmentations for hippocampal subfields were based on previously published criteria [41, 42], and seed‐to‐whole‐brain functional connectivity (FC) analysis was performed using each of the 20 hippocampal subregions (comprising areas including the bilateral parasubiculum, bilateral presubiculum, bilateral subiculum, bilateral CA1, bilateral CA3, bilateral CA4, bilateral HATA, bilateral fimbria, bilateral hippocampal‐tail, bilateral hippocampal‐fissure) as the “seed” region.

A FC map for each seed ROI was generated by calculating the Pearson's correlation coefficient between the time series of each voxel of the whole brain and the meantime course of the voxels within the seed region. Subsequently, Fisher r‐to‐z transformations were applied. The seed‐to‐whole‐brain FC analysis was performed using DPABI toolbox, and we employed a voxel‐wise p‐value threshold of *p* < 0.001 and a cluster threshold of *p* < 0.05, with corrections applied using the Gaussian Random Field (GRF) method.

The ROC curve was generated using randomForest Package to assess the diagnostic performance of Functional connectivity for Severity of MDD. The AUC with a 95% confidence interval (calculated via random forest algorithm.) was reported, and the optimal cut‐off was selected based on the Youden's Index.

### 5.15 Statistical Analysis

Data were analyzed with GraphPad Prism (version 8, San Diego, USA) and R (R4.2.1), with *p* < 0.05 considered statistically significant. All data were expressed as mean ± SEM unless otherwise indicated. Data were analyzed using the non‐parametric Mann‐Whitney test or unpaired Student's *t*‐test where appropriate. Paired *t*‐tests were performed to evaluate changes in metabolites between pre‐ and post‐treatment patients with MDD, and Spearmen's correlation analysis was used to assess the relationship between metabolites and clinical data. Statistical parameters and the exact number of animals employed in each study are reported in the results or figure legends.

## Author Contributions

Y‐G.Y., X‐L.Z. and X.Z. conceived, designed, and supervised the project. X‐Y.C. and T‐P.S. performed all the animal and clinical studies and provided the data. M‐Z.F. and J‐C.Z. participated in the animal experiments and analysis. T‐P.S., G.C., H.Z., D.W., Y.C., and Z.C. were responsible for clinical sample collection and evaluation. Y‐G.Y. supervised the clinical study and provided serum samples. X.Z. provided important inputs in data acquisition and writing. Y‐G.Y. and X‐L.Z. wrote and revised the manuscript with inputs from X.Z. All authors validated and approved the final manuscript.

## Conflicts of Interest

The authors declare no conflicts of interest.

## Supporting information




**Supporting File 1**: advs73486‐sup‐0001‐SuppMat.docx.

**Supporting File 2**: advs73486‐sup‐0002‐TableS1.xlsx.

**Supporting File 3**: advs73486‐sup‐0003‐TableS2.xlsx.

**Supporting File 4**: advs73486‐sup‐0004‐TableS3.xlsx.

## Data Availability

The data that support the findings of this study are available from the corresponding author upon reasonable request.
