# Supplementary material for: Taurocholic Acid Is Associated With Disturbed Functional Connectivity in the Hippocampus of Patients With Depression
Source: Adv Sci (Weinh). 2025 Dec 23;13(13):e08693. doi: 10.1002/advs.202508693 (PMC12955919; doi:10.1002/advs.202508693)
Supplement: Supplementary file 1 — Supporting File 1: advs73486‐sup‐0001‐SuppMat.docx. [file ADVS-13-e08693-s001.docx]

Supporting Information

Taurocholic Acid is Associated with Disturbed Functional Connectivity in the Hippocampus of Patients with Depression

Xiaoying Cai, Taipeng Sun, Mengzhen Feng, Gang Chen, Junchi Zhou, Hong Zhuang, Dan Wang, Ying Chen, Zhen Cheng, Zhi Xu, Xiao Zheng,* Xueli Zhang,* and Yonggui Yuan*


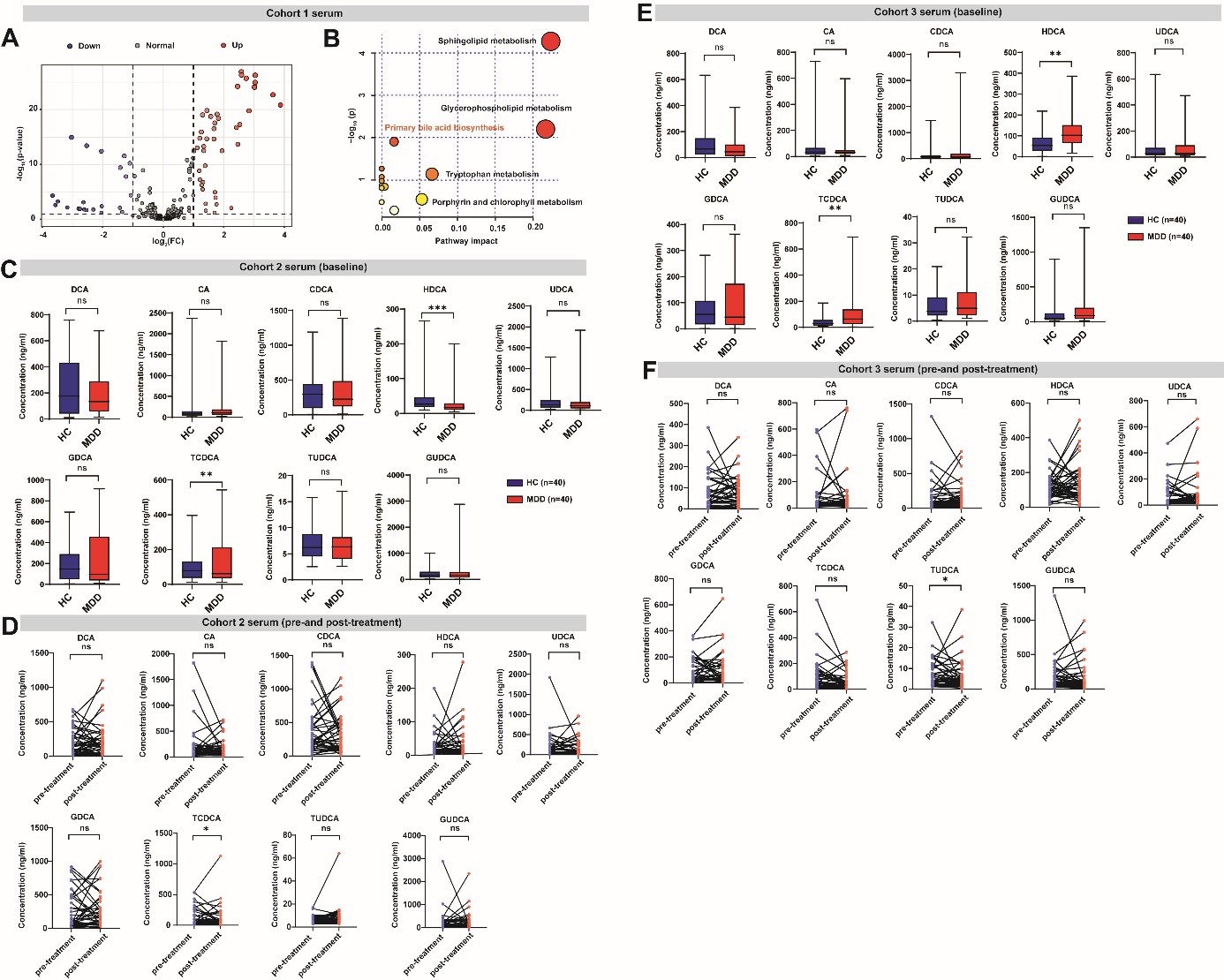


**Figure S1. Serum bile acid levels in the MDD and HC clinical cohorts.**

(A) Volcano plot of serum metabolite profile between MDD and HC groups in cohort 1.

(B) Pathway enrichment analysis of differential metabolites from MDD and HC groups in cohort 2 by using Ingenuity Pathway Analysis (IPA). Red circles denote pathways that have signiﬁcant changes. n=40 per group.

(C) Baseline serum bile acid levels between MDD and HC participants in cohort 2. n=40 per group. Box-and-whisker plots show median and interquartile range (IQR); The whiskers above and below the box show the maximum and minimum.

(D) Paired data plot showing baseline and post-treatment bile acid levels in patients with MDD of cohort 2. n=40 per group.

(E) Baseline serum bile acid levels between MDD and HC participants in cohort 3. n=40 per group. Box-and-whisker plots show median and interquartile range (IQR); The whiskers above and below the box show the maximum and minimum.

(F) Paired data plot showing baseline and post-treatment bile acid levels in patients with MDD of cohort 3. n=40 per group.

**p*<0.05, ***p*<0.01, ****p*<0.001; ns, no significance.Data are analyzed with Rank ANCOVA adjusted with age, gender, and BMI (C, E); or paired Wilcoxon rank-sum test (D, F).


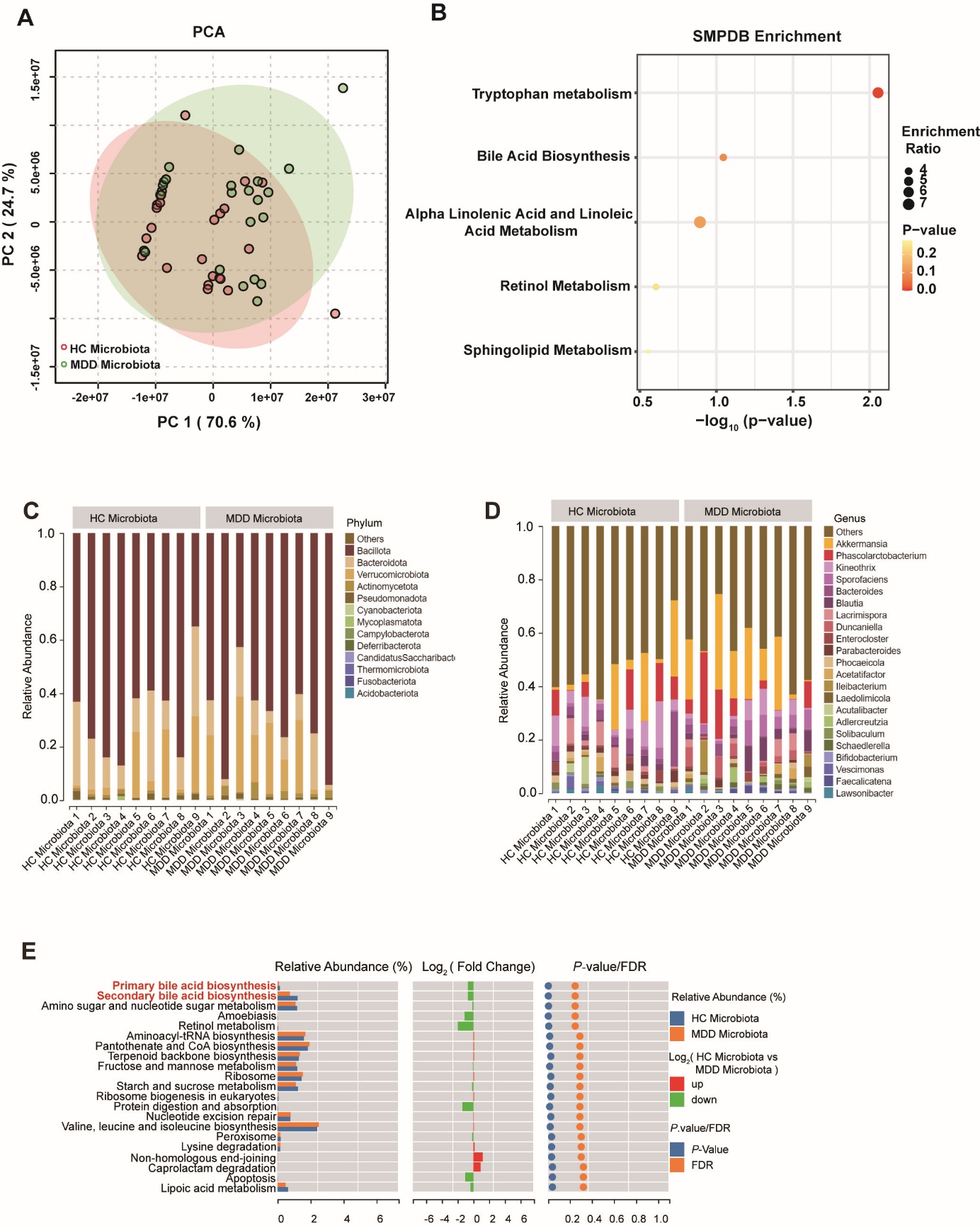


**Figure S2 Metabolomic and microbial analysis in recipient mice**.

(A) Principal component analysis (PCA) of serum metabolites between mice with microbiota derived from patients with MDD (MDD Microbiota) and HC participants (HC Microbiota) by untargeted metabolomic analysis. *p*=0.005, R=0.303. n=24 per group.

(B) SMPDB enrichment analysis of serum metabolite in mice receiving HC and MDD microbiota.

(C) The taxonomic composition distribution of gut microbiome at phylum level. Relative abundance of bacterial phylum in the HC Microbiota and MDD Microbiota group (n=9).

(D) The taxonomic composition distribution of gut microbiome at genus level. Relative abundance of bacterial genus in the HC Microbiota and MDD Microbiota group (n=9).

(E) PICRUST prediction of differentially enriched metabolic pathways in microbiota from the recipient mice. (n=9).


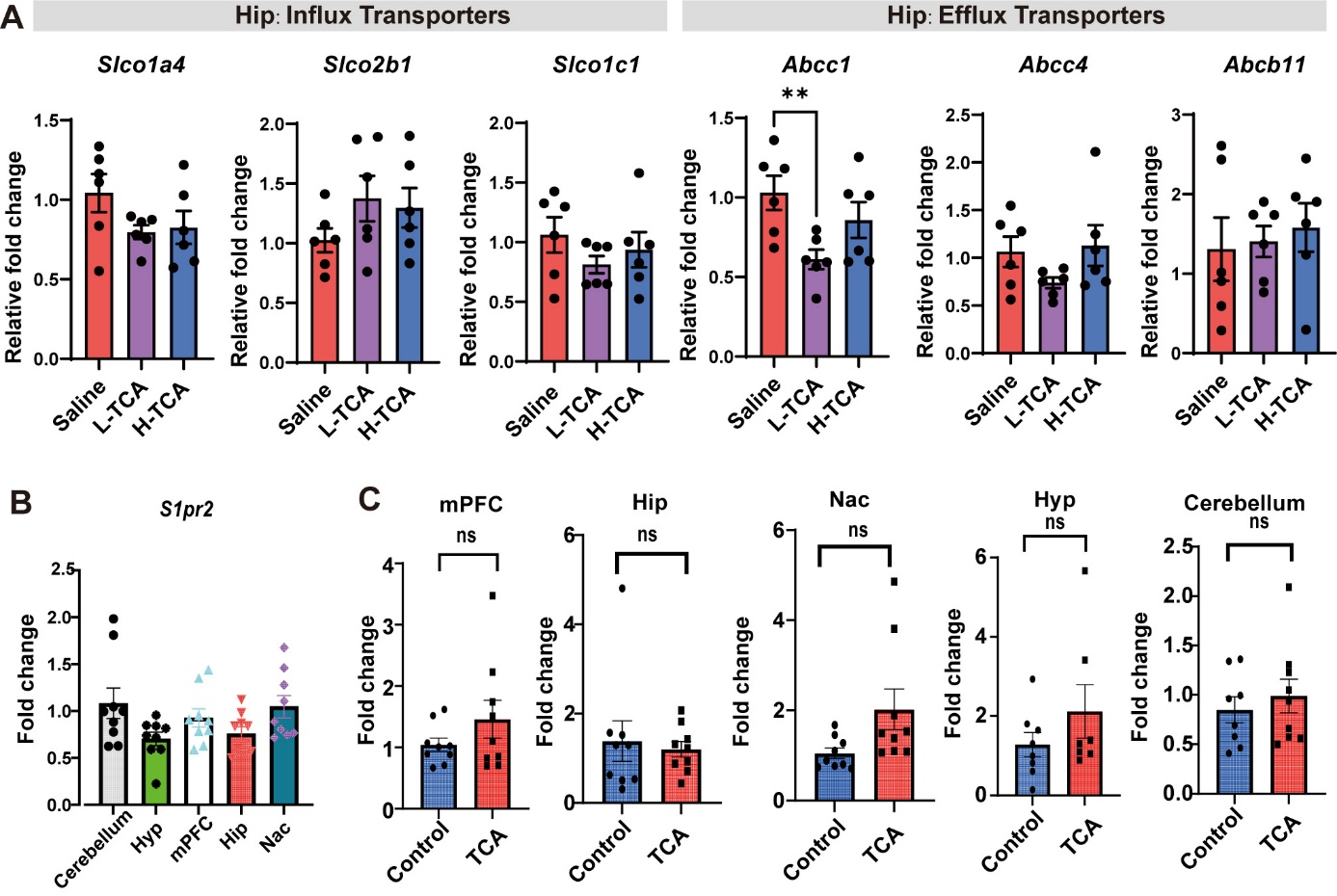


**Figure S3. The impact of TCA on microglial activation, neurogenesis and S1PR2 expression in brain regions**.

1. Relative mRNA expression of influx transporters and efflux transporters of bile acids in hippocampal regions of control and TCA-treated mice. L-TCA (100 mg/kg), H-TCA (200 mg/kg). n=6 mice per group.
2. Relative mRNA expression of *S1rp2* in major brain regions of control mice (n=9).
3. Relative mRNA expression of *S1rp2* in major brain regions of control and TCA-treated mice (n=10).

**p*<0.05, ***p*<0.01, ****p*<0.001; ns, no significance; Data are analyzed by independent samples *t*-test. mPFC, medial prefrontal cortex. HYP, hypothalamus. Hip, hippocampus. Nac, nucleus accumbens.


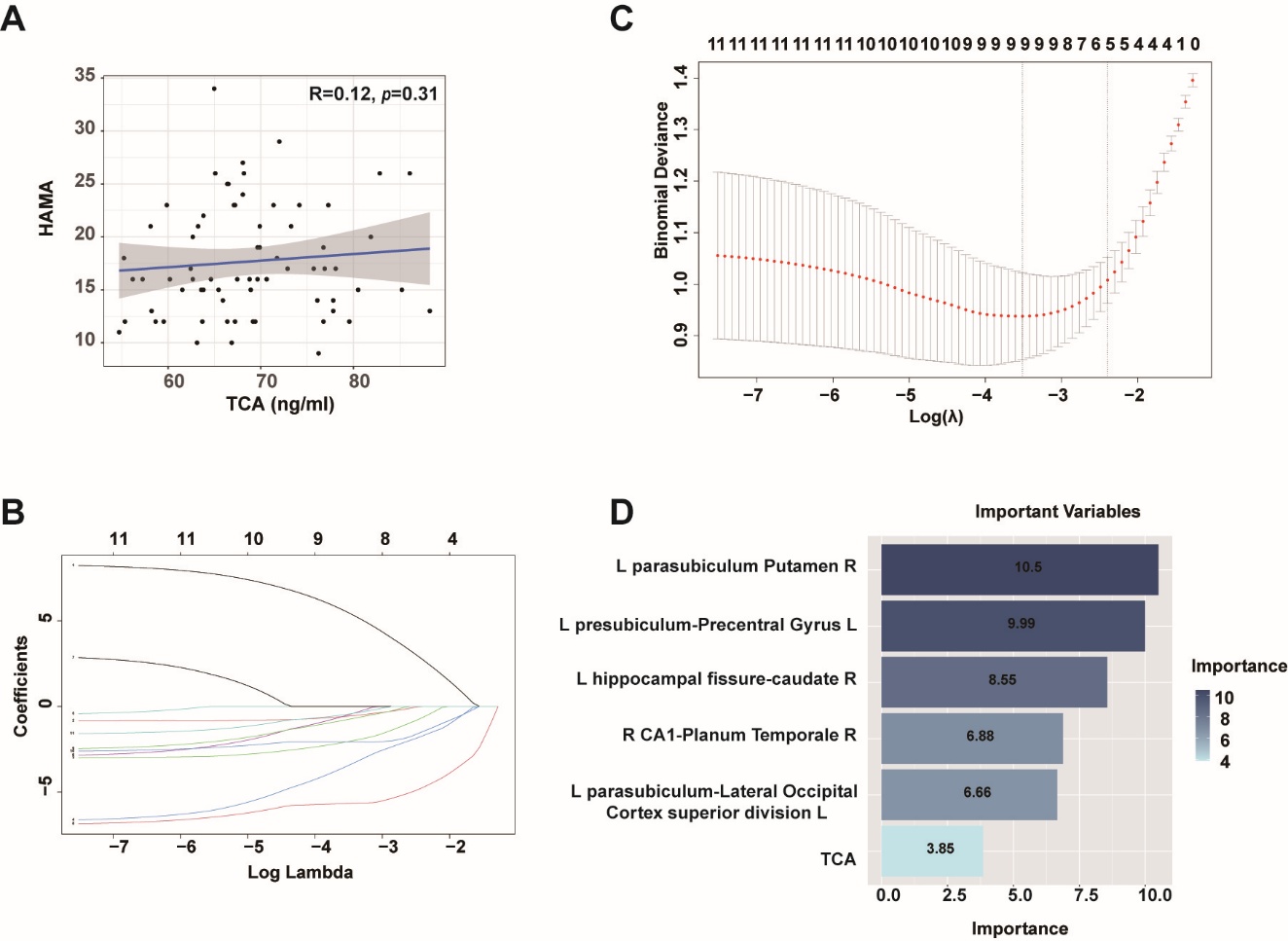


**Figure S4. Feature selection and correlation analysis for the association between serum TCA levels and HAMA scores and feature importance.**

1. Correlation between serum level of TCA and HAMA score in patients with MDD.

(B-C) Feature down-selection performed using LASSO method.

(D) Feature importance from combined TCA and brain characteristics by random forest classification model.

Supplementary tables

Table S1. Demographics and clinical characteristics of cohort 1

|  | **HC (N = 48)** | **MDD (N = 46)** | **t/χ²** | ***p*** |
| --- | --- | --- | --- | --- |
| **Age** | 42.88 ± 8.20 | 39.27 ± 11.69 | 1.748 | 0.084 |
| **BMI** | 23.89 ± 2.94 | 22.62 ± 3.44 | 1.945 | 0.055 |
| **HAMD-24** | 2.02 ± 1.82 | 27.69 ± 4.55 | -36.245 | <0.001 |
| **HAMA** | 1.85 ± 1.53 | 20.33 ± 6.00 | -20.673 | <0.001 |
| **SHAPS** | 13.50 ± 0.98 | 34.72 ± 7.90 | -18.270 | <0.001 |
| **Gender** |  |  | 0.236 | 0.627 |
| Male | 12 (25.00%) | 9 (19.57%) |  |  |
| Female | 36 (75.00%) | 37 (80.43%) |  |  |

Table S2. Demographics and baseline characteristics of cohort 2

| **Characteristic** | **HC (N = 40)** | **MDD (N = 40)** | **t/χ²** | ***p*** |
| --- | --- | --- | --- | --- |
| **Age** | 39.45 ± 12.36 | 43.28 ± 13.83 | -1.304 | 0.196 |
| **BMI** | 23.17 ± 3.42 | 22.24 ± 3.01 | 1.287 | 0.202 |
| **HAMD-24** | 1.65 ± 1.64 | 27.35 ± 5.04 | -21.172 | <0.001 |
| **HAMA** | 1.28 ± 1.46 | 17.28 ± 4.54 | -17.995 | <0.001 |
| **SHAPS** | 22.30 ± 11.94 | 36.25 ± 6.38 | -6.513 | <0.001 |
| **Gender** |  |  | 3.164 | 0.075 |
| Male | 7 (17.50%) | 14 (35.00%) |  |  |
| Female | 33 (82.50%) | 26 (65.00%) |  |  |

Table S3. Demographics and baseline characteristics of cohort 3

|  | **HC (N=40)** | **MDD (N=40)** | **t/χ²** | ***p*** |
| --- | --- | --- | --- | --- |
| **Age** | 38.60 ± 5.37 | 38.70 ± 12.81 | -0.046 | 0.964 |
| **BMI** | 23.01 ± 2.48 | 22.40 ± 4.44 | 0.755 | 0.453 |
| **HAMD-24** | 1.85 ± 2.20 | 26.45 ± 4.56 | -30.710 | <0.001 |
| **HAMA** | 1.00 ± 1.51 | 21.28 ± 3.91 | -30.575 | <0.001 |
| **SHAPS** | 24.80 ± 5.78 | 39.35 ± 7.91 | -9.393 | <0.001 |
| **Gender** |  |  | 0.051 | 0.822 |
| Male | 17 (42.50%) | 18 (45.00%) |  |  |
| Female | 23 (57.50%) | 22 (55.00%) |  |  |

Table S4. Demographics and baseline characteristics of cohort 4

|  | **HC (N = 20)** | **MDD (N = 20)** | **t/χ²** | ***p*** |
| --- | --- | --- | --- | --- |
| **Age** | 37.80 ± 9.83 | 39.35 ± 11.69 | -0.453 | 0.653 |
| **BMI** | 22.86 ± 2.97 | 22.67 ± 3.21 | 0.187 | 0.853 |
| **HAMD-24** | 0.70 ± 1.41 | 27.45 ± 5.22 | -22.092 | <0.001 |
| **HAMA** | 0.65 ± 1.04 | 19.15 ± 5.75 | -14.155 | <0.001 |
| **SHAPS** | 18.60 ± 6.75 | 38.20 ± 7.33 | -8.789 | <0.001 |
| **Gender** |  |  | 0.107 | 0.744 |
| Male | 7 (35.00%) | 8 (40.00%) |  |  |
| Female | 13 (65.00%) | 12 (60.00%) |  |  |

Table S5. Demographics and baseline characteristics of cohort 5 (Huai'an No. 3 hospital)

|  | **HC (N = 20)** | **MDD (N = 20)** | **t/χ²** | ***p*** |
| --- | --- | --- | --- | --- |
| **Age** | 39.65 ± 6.84 | 38.10 ± 15.96 | 0.399 | 0.693 |
| **BMI** | 23.09 ± 2.99 | 22.32 ± 3.21 | 0.789 | 0.435 |
| **HAMD-24** | 1.35 ± 1.49 | 27.80 ± 6.04 | -18.983 | <0.001 |
| **HAMA** | 0.65 ± 1.13 | 21.05 ± 5.57 | -16.029 | <0.001 |
| **SHAPS** | 23.60 ± 5.67 | 47.90 ± 6.52 | -12.568 | <0.001 |
| **Gender** |  |  | 0.102 | 0.749 |
| Male | 9 (45.00%) | 8 (40.00%) |  |  |
| Female | 11 (55.00%) | 12 (60.00%) |  |  |

Table S6. Demographics and baseline characteristics of cohort 6

|  | **HC (N = 64)** | **MDD (N = 69)** | **t/χ²** | ***p*** |
| --- | --- | --- | --- | --- |
| **Age** | 36.78 ± 11.28 | 40.59 ± 11.74 | -1.910 | 0.058 |
| **BMI** | 23.23 ± 3.21 | 22.80 ± 3.42 | 0.752 | 0.454 |
| **HAMD-24** | 1.95 ± 1.72 | 25.88 ± 5.31 | -35.497 | <0.001 |
| **HAMA** | 1.92 ± 1.71 | 17.68 ± 5.28 | -23.479 | <0.001 |
| **SHAPS** | 24.17 ± 5.92 | 35.35 ± 8.64 | -8.750 | <0.001 |
| **Gender** |  |  | 1.572 | 0.210 |
| Male | 19 (29.69%) | 14 (20.29%) |  |  |
| Female | 45 (70.31%) | 55 (79.71%) |  |  |

Table S7. Differences in hippocampal subfields–whole brain connectivity between MDD and healthy controls (HCs).

| **ROI** | **Brain regions** | **X** | **Y** | **Z** | **Cluster size (voxel)** | **Peak intensity** |
| --- | --- | --- | --- | --- | --- | --- |
| **Left**  **hippocampal_fissure** | Caudate_R | 18 | -12 | 24 | 17 | 4.96 |
| **Left parasubiculum** | Central_Opercular_Cortex_L | -45 | -12 | 18 | 15 | -4.08 |
|  | Putamen_R | 33 | 0 | -3 | 21 | -4.95 |
|  | Lateral_Occipital_Cortex_superior_division_L | -45 | -75 | 18 | 19 | -4.56 |
|  | Superior_Temporal_Gyrus_posterior_division_L | -51 | -27 | -3 | 13 | -4.16 |
| **Left presubiculum** | Lateral_Occipital_Cortex_inferior_division_R | 39 | -75 | 3 | 13 | -4.72 |
|  | Middle_Temporal_Gyrus_temporooccipital_part_L | -60 | -51 | 6 | 13 | -4.29 |
|  | Precentral_Gyrus_L | -57 | -3 | 9 | 27 | -4.85 |
| **Right CA1** | Middle_Temporal_Gyrus_temporooccipital_part_L | -60 | -54 | 0 | 17 | -4.47 |
|  | Planum_Temporale_R | 63 | -18 | 6 | 34 | -4.69 |
| **Right hippocampal_fissure** | Middle_Temporal_Gyrus_posterior_division_R | 66 | -30 | -6 | 14 | -3.88 |

Center (MNI): X, Y, Z. Abbreviations: MNI = Montreal Neurological Institute. ROI=regions of interest, L=left, R=right.

Table S8. Patient information of fecal microbiota transplant donors

| ID | Gender | Age  (year) | Duration of illness (month) | HAMD-24  score | HAMA score | SHAPS score |
| --- | --- | --- | --- | --- | --- | --- |
| MDD1 | female | 53 | 24 | 27 | 24 | 39 |
| MDD2 | female | 50 | 4 | 24 | 13 | 41 |
| MDD3 | male | 34 | 48 | 35 | 17 | 42 |
| MDD4 | female | 52 | 7 | 50 | 24 | 56 |
| HC1 | male | 39 | - | 0 | 0 | 28 |
| HC2 | female | 37 | - | 7 | 2 | 18 |
| HC3 | female | 32 | - | 2 | 1 | 21 |
| HC4 | male | 46 | - | 3 | 1 | 23 |

Table S9. Primers for qPCR analysis.

| Gene | Forward (5'-3') | Reverse (5'-3') |
| --- | --- | --- |
| Gapdh | CTGCCCAGAACATCATCCCT | TGAAGTCGCAGGAGACAACC |
| S1pr2 | ATGGGCGGCTTATACTCAGAG | GCGCAGCACAAGATGATGAT |
| Slco1a4 | AGATCCTGCAGAAATGAAGCTC | AAATGACCAACTGTGTGACAGG |
| Slco2b1 | GCCAGAAGGAGGCATCAACT | TTAAAGGCTCGTGCTGGGAG |
| Slco1c1 | CAGCATCTCTCCGTGCTACC | AGGAGCTGGAATCCCCTACA |
| Abcc1 | GCGCTGTCTATCGTAAGGCT | AGAGGGGCTGACCAGATCAT |
| Abcc4 | TCCCTTGTTCTGGCGAAGAC | CGAAGACGATGACTCCCTCG |
| Abcb11 | CAAACGGAACAAGCTGTGGG | TCCTTATCTGACGAGCCCCA |
